# Withdrawal: Reactive oxygen species (ROS) mediate p300-dependent STAT1 protein interaction with peroxisome proliferator-activated receptor (PPAR)-γ in CD36 protein expression and foam cell formation

**DOI:** 10.1016/j.jbc.2022.102152

**Published:** 2022-06-28

**Authors:** Sivareddy Kotla, Gadiparthi N. Rao

This article has been withdrawn by the authors. The authors and the journal conclude that the images between and within Figures 2D and 3D were reused. Specifically, the image “pcDNA” in Figure 3D was a duplication of the image “GW9662 + Vehicle” in Figure 2D. Similarly, the image “p300WT + 15(S)-HETE” in Figure 3D was captured from the well “PFS1YF + 15(S)-HETE” in Figure 2D. The image “p300WT” in Figure 3D was captured from the well “pcDNA + 15(S)-HETE” in Figure 3D itself. Additional potential internal reuse is in Figure 2D, STAT1(WT) + 15(S)-HETE” and Figure 2D, “PFS1YF + 15(S)-HETE”. The authors state that these image duplications do not affect the conclusions of the paper. The authors apologize for this oversight.

